# Structuring and extracting knowledge for the support of hypothesis generation in molecular biology

**DOI:** 10.1186/1471-2105-10-S10-S9

**Published:** 2009-10-01

**Authors:** Marco Roos, M Scott Marshall, Andrew P Gibson, Martijn Schuemie, Edgar Meij, Sophia Katrenko, Willem Robert van Hage, Konstantinos Krommydas, Pieter W Adriaans

**Affiliations:** 1 Informatics Institute, University of Amsterdam, Amsterdam, 1098 SJ The Netherlands; 2 Swammerdam Institute for Life Science, University of Amsterdam, Amsterdam, 1018 WB The Netherlands; 3 BioSemantics group, Erasmus University of Rotterdam, Rotterdam, 3000 DR The Netherlands; 4 Business Informatics, Faculty of Sciences, Vrije Universiteit, Amsterdam, 1081 HV The Netherlands

**Keywords:** Text Mining, Biological Model, Conditional Random Field, Entity Recognition, Knowledge Extraction

## Abstract

**Background:**

Hypothesis generation in molecular and cellular biology is an empirical process in which knowledge derived from prior experiments is distilled into a comprehensible model. The requirement of automated support is exemplified by the difficulty of considering all relevant facts that are contained in the millions of documents available from PubMed. Semantic Web provides tools for sharing prior knowledge, while information retrieval and information extraction techniques enable its extraction from literature. Their combination makes prior knowledge available for computational analysis and inference. While some tools provide complete solutions that limit the control over the modeling and extraction processes, we seek a methodology that supports control by the experimenter over these critical processes.

**Results:**

We describe progress towards automated support for the generation of biomolecular hypotheses. Semantic Web technologies are used to structure and store knowledge, while a workflow extracts knowledge from text. We designed minimal proto-ontologies in OWL for capturing different aspects of a text mining experiment: the biological hypothesis, text and documents, text mining, and workflow provenance. The models fit a methodology that allows focus on the requirements of a single experiment while supporting reuse and posterior analysis of extracted knowledge from multiple experiments. Our workflow is composed of services from the 'Adaptive Information Disclosure Application' (AIDA) toolkit as well as a few others. The output is a semantic model with putative biological relations, with each relation linked to the corresponding evidence.

**Conclusion:**

We demonstrated a 'do-it-yourself' approach for structuring and extracting knowledge in the context of experimental research on biomolecular mechanisms. The methodology can be used to bootstrap the construction of semantically rich biological models using the results of knowledge extraction processes. Models specific to particular experiments can be constructed that, in turn, link with other semantic models, creating a web of knowledge that spans experiments. Mapping mechanisms can link to other knowledge resources such as OBO ontologies or SKOS vocabularies. AIDA Web Services can be used to design personalized knowledge extraction procedures. In our example experiment, we found three proteins (NF-Kappa B, p21, and Bax) potentially playing a role in the interplay between nutrients and epigenetic gene regulation.

## Background

**Figure 1:**
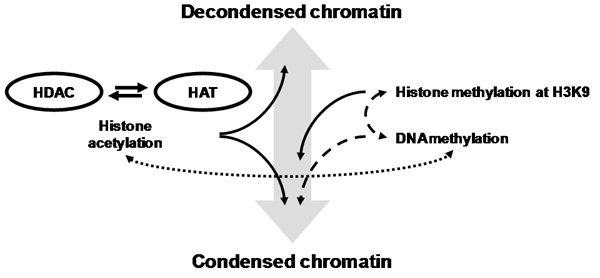
**Cartoon model for the mechanism of chromatin condensation and decondensation**. Models for condensation and decondensation of chromatin, a determinant of transcriptional activity, involves enzymes for histone acetylation (HAT) and histone deacetylase (HDAC), DNA methylation, and methylation of histone H3K9 [47]. Cartoon representations are a typical means for scientific discourse for molecular biologists.

In order to study a biomolecular mechanism such as epigenetic gene control (Figure 1) and formulate a new hypothesis, we usually integrate various types of information to distil a comprehensible model. We can use this model to discuss with our peers before we test the model in the laboratory or by comparison to available data. A typical hypothesis is based on one's own knowledge, interpretations of experimental data, the opinions of peers, and the prior knowledge that is contained in literature. Many Web resources are available for molecular biologists to access available knowledge, of which Entrez PubMed, hosted by the US National Center for Biotechnology Information (NCBI), is probably the most used by molecular biologists. The difficulty of information retrieval from literature reveals the scale of today's information overload: over 17 million biomedical documents are now available from PubMed. Also considering the knowledge that did not make it to publication or that is stored in various types of databases and file systems, many scientists find it increasingly challenging to ensure that all potentially relevant facts are considered whilst forming a hypothesis. Support for extracting and managing knowledge is therefore a general requirement. Developments in the area of the Semantic Web and related areas such as information retrieval are making it possible to create applications that will support the task of hypothesis generation. First, RDF and OWL provide us with a way to represent knowledge in a machine readable format that is amenable to machine inference [1, 2]. Ontologies have become an important source of knowledge in molecular biology. Many ontologies have been created and many types of application have become possible [3], with the life sciences providing a key motivation of addressing the information management problem that arises from high throughput data collection [4, 5]. A downside to the popularity of bio-ontologies is that their number and size have become overwhelming when attempting to discover the best representation for one's personal hypothesis. Moreover, building a biological ontology is usually associated with a community effort where consensus is sought for clear descriptions of biological phenomena [6]. The question arises how an experimental biologist/bioinformatician can apply Semantic Web languages when the primary aim is not to build a comprehensive ontology for a community, but to represent a personal hypothesis for a particular biomolecular mechanism. Therefore, we explored an approach to semantic modeling that emphasizes the creation of a personal model within the scope of one hypothesis, but without precluding integration with other ontologies. Secondly, information retrieval and information extraction techniques can be used to elucidate putative knowledge to consider for a hypothesis by selecting relevant data and recognizing biological entities (e.g. protein names) and relations in text [5, 8]. For instance, tools and algorithms have been developed that match predefined sets of biological terms [7, 8], or that use machine learning algorithms to recognize entities and extract relations based on their context in a document [9]. These techniques can also be used to extend an ontology [10, 11]. Several tools exist for text mining (See, for instance [8]), but for a methodology to be attractive to practitioners of experimental molecular biology we would like a method that is more directly analogous to wet laboratory experimentation. Workflow management systems offer a platform for in silico experimentation [12–14] where, for example, data integration [5, 15], and systematic large-scale analysis [16] have been implemented. Workflows can also be shared on the web such as accomplished in myExperiment [17]. In a workflow, the steps of a computational experiment are carried out by individual components for which Web Services provide a common communication protocol [18]. We adopted the workflow paradigm for the design and execution of a reusable knowledge extraction experiment. The main services in the workflow are from the 'Adaptive Information Disclosure Application' toolkit (AIDA) that we are developing for knowledge management applications [19] and this document). The output enriches a knowledge base with putative biological relations and corresponding evidence. The approach is not limited to text mining but can be applied to knowledge extracted during any computational experiment. The advantage of routinely storing extracted knowledge is that it enables us to perform posterior analysis across many experiments.

## Results

We present the methodology in the following order: 1) a description of representing prior knowledge through proto-ontologies; 2) extension of the proto-ontologies by a workflow that adds instances to a semantic repository preloaded with the proto-ontologies; 3) a description of how to query the knowledge base; 4) a description of the toolkit that we use for knowledge extraction and knowledge management. Data and references are accessible from pack 58 on myExperiment.org [20].

### Model representation in OWL

#### Different types of knowledge

Step one of our methodology is to define machine readable 'proto-ontologies' to represent our biological hypothesis within the scope of an experiment. The experiment in this case is a procedure to extract protein relations from literature. Our approach is based on the assumption that knowledge models can grow with each experiment that we or others perform. Therefore, we created a minimal OWL ontology of the relevant biological domain entities and their biological relations for our knowledge extraction experiment. The purpose of the experiment is to populate (enrich) the proto-ontologies with instances derived from literature. We also modeled the evidence that led to these instances. For instance, the process by which a protein name was found and in which document it was found. We find a clash between our intention of enriching a biological model, and the factual observations of a text mining procedure such as 'term', 'interaction assertion', or 'term collocation'. For example, it is obvious that collocation of the terms 'HDAC1' and 'p53' in one abstract does not necessarily imply collocation of the referred proteins in a cell. In order to avoid conflation of knowledge from the different stages of our knowledge extraction process, we purposefully kept distinct OWL models. This lead to the creation of the following models that will be treated in detail below:

❑ Biological knowledge for our hypothesis (Protein, Association)

❑ Text (Terms, Document references)

❑ Knowledge extraction process (Steps of the procedure)

❑ Extraction procedure implementation (Web Service and Workflow runs)

❑ Mapping model to integrate the above through references.

❑ Results (Instances of extracted terms and relations)

#### Biological model

**Figure 2:**
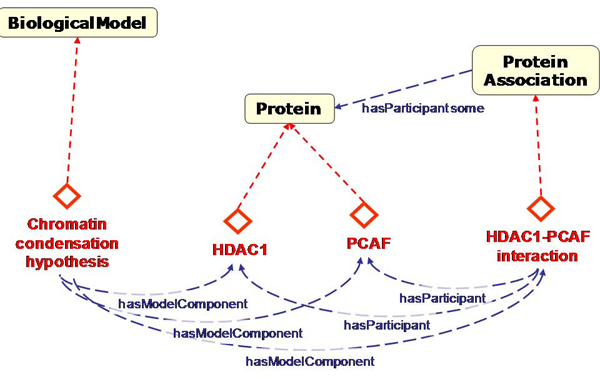
**Graphical representation of the biological domain model in OWL and example instances**. This proto-ontology contains classes for instances that may be relevant in hypotheses about chromatin (de)condensation. HDAC1 and PCAF are example instances representing proteins implied in models about this process and known to interact. In this and following figures, red diamonds represent instances, dashed arrows connected to diamonds represent instance-of relations and blue dashed arrows represent properties between classes or instances. Inverse relations are not shown for clarity. Protein Association represents the reified relation in which two (or more) proteins participate. Instances of 'BiologicalModel' represent an abstraction of a biological hypothesis that can be partially represented by user queries, proteins provided by the user, and proteins discovered by text mining.

For the biological model, we started with a minimal set of classes designed for hypotheses about proteins and protein-protein associations (Figure 2). This model contains classes such as 'Protein', 'Interaction' and 'Biological Model'. We regard instances in the biological model as interpretations of certain observations, in our case, of text mining results. We also do not consider instances of these classes as biological facts; they are restricted to a hypothetical model in line with common practice in experimental biology. The evidence for the interpretation is important, but it is not within the scope of this model. In the case of text mining, evidence is modeled by the text, text mining, and implementation models.

#### Text model

**Figure 3:**
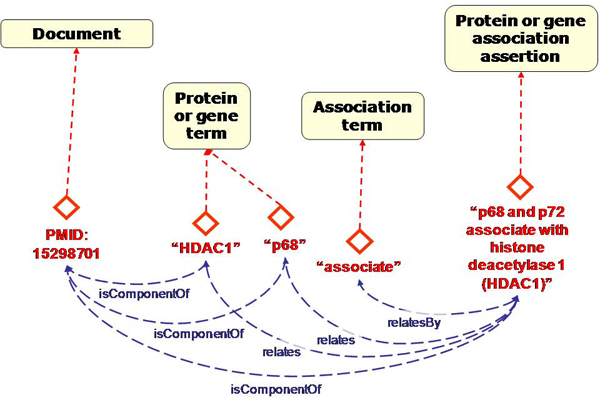
**Graphical representation of proto-ontology for entities in text and example instances**. This proto-ontology contains classes of instances for documents, terms, and statements found in the text of the documents. The latter relation is represented by 'component of' properties. The instances represent concrete observations in text. Properties such as 'relates' and 'relatesBy' represent their interrelations. Example instances are shown for protein names 'HDAC1' and 'p68' and an assertion suggesting a relation between these two proteins.

A model of the structure of documents and statements therein is less ambiguous than the biological model, because we can directly inspect concrete instances such as (references to) documents or pieces of text (Figure 3). We can be sure of the scope of the model and we can be clear about the distinction between classes and instances because we computationally process the documents. This model contains classes for documents, protein or gene names, and mentions of associations between proteins or genes.

#### Text mining model

**Figure 4:**
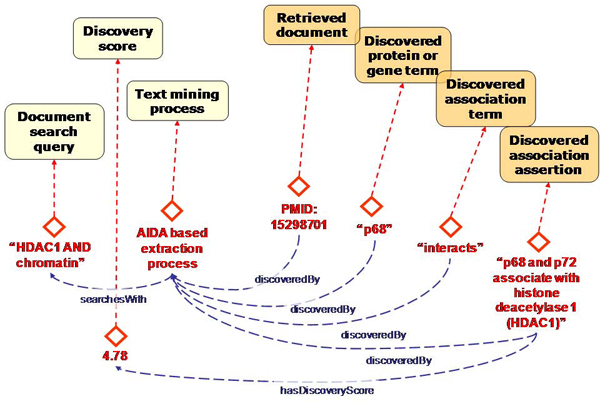
**Graphical representation of the proto-ontology for the text mining process**. This proto-ontology contains the classes for instances of the processes by which a knowledge extraction experiment is performed. The darker coloured classes represent restriction classes for instances that have at least one 'discoveredBy' property defined.

Next, we created a model for the knowledge extraction process. This model serves to retrieve the evidence for the population of our biological model (Figure 4). It contains classes for information retrieval and information extraction such as 'collocation process' and properties such as 'discovered by'. We also created classes to contain text mining specific information such as the likelihood of terms being found in the literature. This allows us to inspect the uncertainty of certain findings. Because any procedure could be implemented in various ways, we created a separate model for the implementation artifacts.

#### Workflow model

For more complete knowledge provenance, we also created a model representing the implementation of the text mining process as a workflow of (AIDA) Web Services. Example instances are (references to) the AIDA Web Services, and runs of these services. Following the properties of these instances we can retrace a particular run of the workflow.

#### Mapping model

**Figure 5:**
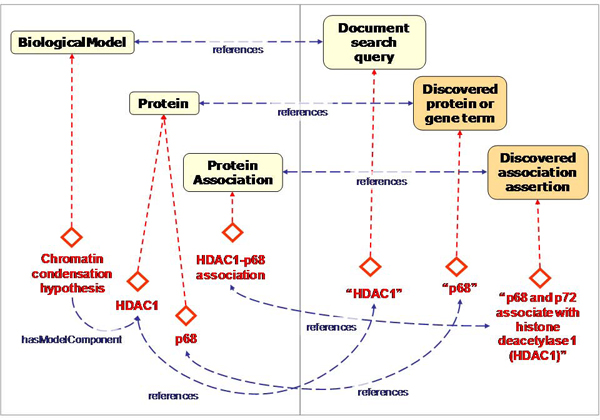
**Graphical representation of the proto-ontology containing the mapping properties between the biological, text, and text mining models**. The 'reference' properties connect the concrete observations captured in the text model with the model representations in the biological model. For instance, the discovered protein name 'HDAC1' in the text mining model refers to the protein labelled 'HDAC1' that is a component of an instance representing a chromatin condensation hypothesis.

At this point, we have created a clear framework for the description of our biological domain and the documents and the text mining results as instances in our text and text mining ontologies. The next step is to relate the instances in the various models to the biological domain model. Our strategy is to initially keep the domain model simple at the class and object property level, and to map sets of instances from our results to the domain model. For this, we created an additional mapping model that defines reference properties between the models (Figure 5). This allows us to see that an interaction between the proteins labeled 'p68' and 'HDAC1' in our hypothetical model is referred to by a mention of an association between the terms 'p68' and 'HDAC1', with a likelihood score that indicates how remarkable it is to find this combination in literature.

**Figure 6:**
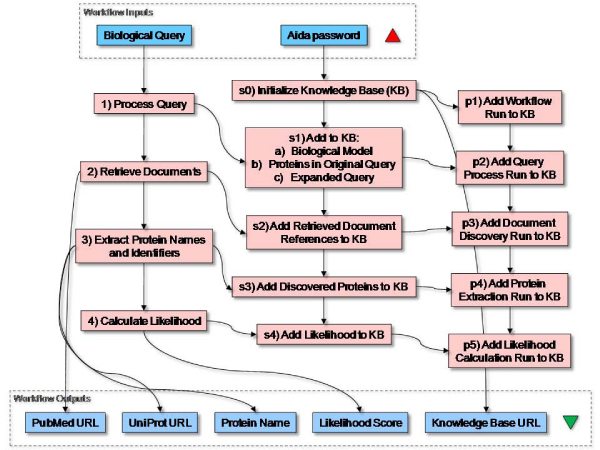
**Knowledge extraction workflow**. The knowledge extraction workflow has three parts. The left part executes the steps of a basic text mining procedure: (i) extract protein names from the user query and add synonyms using the BioSemantics synonym service, (ii) retrieve documents from MedLine with the AIDA document search service, (iii) extract proteins with the AIDA named entity recognition service, (iv) calculate a ranking score for each discovery. The middle workflow converts the results from the text mining workflow to RDF using the biological model and the text model as template. The workflow on the right-side creates execution-level instances for the workflow components and couples these to the instances created in the middle workflow. The blue rectangles represent inputs and outputs. The pink rectangles represent sub-workflows.

In summary, we have created proto-ontologies that separate the different views on biomolecular knowledge derived from literature by a text mining experiment. We can create instances in each view and their interrelations (Figure 6). This allows us to trace the experimental evidence for knowledge contained in the biological model. In a case of text mining such as ours, evidence is modeled by the document, text mining, and workflow models. A different type of computational experiment would require other models and new mappings to represent evidence.

### Knowledge extraction experiment

The proto-ontologies form the basis of our knowledge base. They provide the initial templates for the knowledge that we wish to be able to interrogate in search of new hypotheses. The next step is to populate the knowledge base with instances. At the modeling stage we already anticipated that our first source of knowledge would be literature, and that we would obtain instances by text mining. An element of our approach is to regard knowledge extraction procedures as 'computational experiments' analogous to a wet laboratory experiments. We therefore used the workflow paradigm to design the protocol of our text mining experiment, here with the workflow design and enactment tool Taverna [13, 21]. A basic text mining workflow consists of the following steps: (i) Retrieve relevant documents from MedLine, in particular their abstracts, (ii) Extract protein names from the retrieved abstracts, and (iii) Present the results for inspection. We implemented the text mining process as a workflow (Figure 6). We added an additional sub-workflow to process the input query in order to extract known protein names from the input query and expand the query with synonyms for known protein names. For this, we employed a Web Service that provides UniProt identifiers and synonyms for human, rat and mouse gene names. These were derived from a combination of several public databases [22]. The query is first split into its individual terms with a service from the AIDA Toolkit that wraps the Lucene tokenizer, and then all the terms (tokens) from the original query are checked for having a UniProt identifier by which they are identified as referring to a known protein. The sub-workflow makes the synonyms, UniProt identifiers, and the expanded query available for the rest of the workflow. The expanded query is the input for the next sub-workflow: document retrieval. We applied the document search service from the AIDA Toolkit, parameterized to use the regularly updated MedLine index that is stored on our AIDA server and updated daily. The output of this retrieval service is an xml document that contains elements of the retrieved documents, such as the PubMed identifier, title, abstract, and the authors. We then extract titles and abstracts for the next sub-workflow: i.e. protein name recognition. Sub-workflow 3 employs the AIDA Web Service 'applyCRF' to recognize protein (or gene) names in text. This service wraps a machine learning method based on the 'conditional random fields' approach [23]. In this case it uses a recognition model trained on protein/gene names. We added the aforementioned UniProt service again to mark the extracted results as genuine human, rat, or mouse protein/gene names. In a number of cases the workflow produced more than one identifier for a single protein name. This is due to the ambiguity in gene and protein names. For instance, Tuason *et al.* reported 6.6% ambiguous occurrences of mouse gene names in text, and percentages ranging from 2.4% to 32.9% depending on the organism [24]. The final step of our text mining procedure was to calculate a likelihood score for the extracted proteins to be found in documents retrieved through the expanded input query. We used a statistical method where the likelihood of finding a document with input query (q) and discovered protein name (d) is calculated by: , in which *Q*, *D*, and *QD* are the frequencies of documents containing q, d, and q *and* d, respectively; *QD*_*exp*_is the expected frequency of documents containing q and d assuming that their co-occurrence is a random event; *N* is the total number of documents in MedLine.

**Figure 7:**
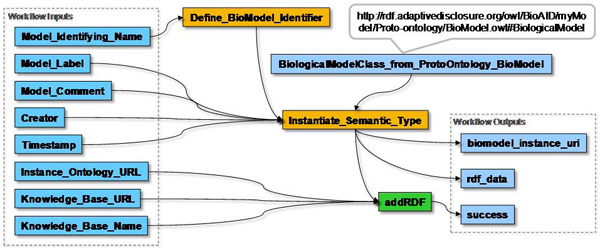
**Example RDF conversion workflow**. This workflow creates an OWL instance for a biological hypothesis in RDF 'N3' format, and adds the RDF triples to the AIDA knowledge base with the 'addRDF' operation of the AIDA repository Web Service. The actual conversion is performed in the Java Beanshell 'Instantiate_Semantic_Type' of which the code is shown at the bottom. The sub-workflow has the hypothesis instance as output for use by other sub-workflows in the main workflow.

In parallel to the part of the workflow that performs the basic text mining procedure, we designed a set of 'semantic' sub-workflows to convert the text mining results to instances of the proto-ontologies and add these instances to the AIDA knowledge base, including their interrelations (steps s *N* in Figure 6). The first step of this procedure is to initialize this knowledge base after which the proto-ontologies are loaded into the knowledge base, and references to the knowledge base are available for the rest of the workflow. The next step is to add instances for the following entities to the knowledge base: 1) the initial biological model/hypothesis, 2) the original input query, 3) the protein names it contains, and 4) the expanded query. We assumed that the input query and the proteins mentioned therein partially represent the biological model; each run of the workflow creates a new instance of a biological model unless the input query is exactly the same as in a previous experiment. Figure 7 illustrates the creation of an instance of a biological model and its addition to the knowledge base, including the details for creating the RDF triples in Java. All the semantic sub-workflows follow a similar pattern (data not shown). The following sub-worfklow adds instances for retrieved documents to the knowledge base; it only uses the PubMed identifier. The sub-workflow that adds discovered proteins is critical to our methodology. It creates protein term instances from protein names in the Text ontology to which it also adds the collocation relations with the original query a and a 'discovered_in' relation with the document it was discovered in. In addition, it creates protein instances in the BioModel ontology and a biological association relation to the proteins found in the input query. Between term and protein instances in the different ontologies it creates reference relations. As a result, our knowledge base is populated with the discoveries of the text mining procedure and their biological interpretations still linked with the knowledge they are interpretations of. The final sub-workflow adds the calculated likelihood scores as a property of the protein terms in the knowledge base. Finally, to be able to retrieve more complete evidence from the knowledge base, we extended our models and workflow to accommodate typical provenance data (not shown). We created an ontology with classes for Workflow runs and Web Service runs. Using the same semantic approach as above we were able to store instances of these runs, including the date and time of execution.

### Querying the knowledge base

**Figure 8:**
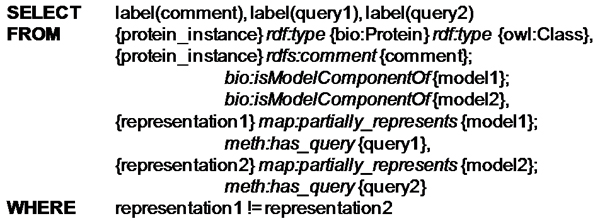
**Pseudo RDF query for extracting proteins related to two hypotheses**. RDF queries are pattern matching queries. This query returns proteins that were found by mining for relations with two different hypotheses represented by two different user queries. The result is a table of protein descriptions and the two user queries.

**Figure 9:**
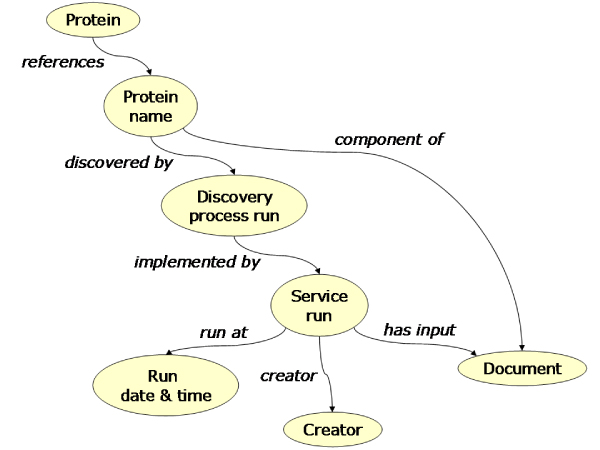
**Graphical representation of a 'chain of evidence query'**. This RDF query matches patterns in the RDF graph created by the knowledge extraction workflow. The result is a table of protein identifiers, protein names, the process by which the proteins were found, the service that implemented this process, the date and time it was run, its creator, and the document that the service used as input and of which the protein name was a component.

The result of running the workflow is that our knowledge base is enriched with instances of biological concepts and relations between those instances that can also tell us why the instances were created. We can examine the results in search of unexpected findings or we can examine the evidence for certain findings, for instance by examining the documents in which some protein name was found. An interesting possibility is to explore relations between the results of computational experiments that added knowledge to the knowledge base. To prove this concept we ran the workflow twice, first with "HDAC1 AND chromatin" as input, and then with "(Nutrition OR food) AND (chromatin OR epigenetics) AND (protein OR proteins)" as input. We were then able to retrieve three proteins that are apparently shared between the two biological models (see Figure 8 for the RDF query): NF-kappaB (UniProt ID P19838), p21 (UniProt ID P38936), and Bax (UniProt ID P97436). If we would like to investigate the evidence by which these proteins were discovered we designed a query that traces the chain of evidence (Figure 9). It retrieves the process by which the name of the protein was found, the service by which the process was implemented and its creator, the document from MedLine in which the protein name was discovered, and the time when this discovery service was run. For example, NF-KappaB was found on the 18^th^ of November 2008 in a paper with PubMed identifier 17540846, by a run of the 'AIDA CRF Named Entity Recognition service' based on 'conditional random fields trained on protein names', created by Sophia Katrenko.

### The AIDA Toolkit for knowledge extraction and knowledge management

The methodology that we propose enables a 'do-it-yourself' approach to extracting knowledge that can support hypothesis generation. To support this approach, we are developing an open source toolkit called Adaptive Information Disclosure Application (AIDA). AIDA is a generic set of components that can perform a variety of tasks related to knowledge extraction and knowledge management, such as perform specialized search on resource collections, learn new pattern recognition models, and store knowledge in a repository. W3C standards are used to make data accessible and manageable with Semantic Web technologies such as OWL, RDF(S), and SKOS. AIDA is also based on Lucene and Sesame. Most components are available as web services and are open source under an Apache license. AIDA is composed of three main modules: Search, Learning, and Storage.

#### Search – the information retrieval module

AIDA provides components which enable retrieval from a set of documents given a query, similar to popular search engines such as Google, Yahoo!, or PubMed. To make a set of documents (a corpus) searchable, an 'index' needs to be created first [25]. For this the AIDA's configurable Indexer can be used. The Indexer and Search components are built upon Apache Lucene, version 2.1.0 [26], and, hence, indexes or other systems based on Lucene can easily be integrated with AIDA. The Indexer component takes care of the preprocessing (the conversion, tokenization, and possibly normalization) of the text of each document as well as the subsequent index generation. Different fields can be made retrievable such as title, document name, authors, or the entire contents. The currently supported document encodings are Microsoft Word, Portable Document Format (PDF), MedLine, XML, and plain text. The so-called "DocumentHandlers" which handle the actual conversion of each source file are loaded at runtime, so a handler for any other proprietary document encoding can be created and used instantly. Because Lucene is used as a basis, a plethora of options and/or languages are available for stemming, tokenization, normalization, or stop word removal which may all be set on a per-field, per-document type, or per-index basis using the configuration. An index can currently be constructed using either the command-line, a SOAP webservice (with the limitation of 1 document per call), or using a Taverna plugin.

#### Learning – the machine learning module

AIDA includes several components which enable information extraction from text data in the Learning module. These components are referred to as learning tools. The large community working on the information extraction task has already produced numerous data sets and tools to work with. To be able to use existing solutions, we incorporated some of the models trained on the large corpora into the named entity recognition web service NERecognizerService. These models are provided by LingPipe[27] and range from the very general named entity recognition (detecting locations, person and organization names) to the specific models in the biomedical field created to recognize protein names and other bio-entities. We specified several options for input/output, which gives us an opportunity to work with either text data or the output of the search engine Lucene. We also offer the LearnModel web service whose aim is to produce a model from annotated text data. A model is based on the contextual information and uses learning methods provided by Weka [28] libraries. Once such a model is created, it can be used by the TestModel web service to annotate texts in the same domain. In this paper we use an AIDA service that applies a service for an algorithm that uses sequential models, such as conditional random fields (CRFs)/CRFs have an advantage over Hiddem Markov Models because of their ability to relax the independence assumption by defining a conditional probability distribution over label sequences given an observation sequence. We used CRFs to detect named entities in several domains like acids of various lengths in the food informatics field or protein names in the biomedical field [9].

Named entity recognition constitutes only one subtask in information extraction. Relation extraction can be viewed as the logical next step after the named entity recognition is carried out [29]. This task can be decomposed into the detection of named entities, followed by the verification of a given relation among them. For example, given extracted protein names, it should possible to infer whether there is any interaction between two proteins. This task is accomplished by the RelationLearner web service. It uses an annotated corpus of relations to induce a model, which consequently can be applied to the test data with already detected named entities. The RelationLearner focuses on extraction of binary relations given the sentential context. Its output is a list of the named entities pairs, where the given relation holds.

The other relevant area for information extraction is detection of the collocations (or n-grams in the broader sense). This functionality is provided by the CollocationService which, given a folder with text documents, outputs the n-grams of the desired frequency and length.

#### Storage – the metadata storage module

AIDA includes components for the storage and processing of ontologies, vocabularies, and other structured metadata in the Storage module. The main component, also for the work described in this paper, is RepositoryWS, a service wrapper for Sesame – an open source framework for storage, inferencing and querying of RDF data on which most of this module's implementation is based [30, 31]. ThesaurusRepositoryWS is an extension of RepositoryWS that provides convenient access methods for SKOS thesauri. The Sesame RDF repository offers an HTTP interface and a Java API. In order to be able to integrate Sesame into workflows we created a SOAP service that gives access to the Sesame Java API. We accommodate for extensions to other RDF repositories, such as the HP Jena, Virtuoso, Allegrograph repositories or future versions of Sesame, by implementing the Factory design pattern.

#### Complementary services from BioSemantics applications

One of the advantages of a workflow approach is the ability to include services created elsewhere in the scientific community ('collaboration by Web Services'). For instance, in our BioAID workflows operations are used for query expansion and validation of protein names by UniProt identifiers. AIDA is therefore complemented by services derived from text mining applications such as Anni 2.0 from the BioSemantics group [32]. The 'BioSemantics' group is particularly strong in disambiguation of the names of biological entities such as genes/proteins, intelligent biological query expansion (manuscript in preparation), and provision of several well known identifiers for biological entities through carefully compiled sets of names and identifiers around a biological concept.

#### User interfaces for AIDA

In addition to RDF manipulation within workflows as described in this document, several examples of user interactions have been made available in AIDA clients such as HTML web forms, AJAX web applications, and a Firefox toolbar. The clients access RepositoryWS for querying RDF through the provided Java Servlets. The web services in Storage have recently been updated from the Sesame 1.2 Java API to the Sesame 2.0 Java API. Some of the new features that Sesame 2.0 provides, such as SPARQL support and named graphs, are now being added to our web service API's and incorporated into our applications.

## Discussion

Our methodology for supporting the generation of a hypothesis about a biomolecular mechanism is based on a combination of tools and expertise from the fields of Semantic Web, e-Science, information retrieval, and information extraction. This novel combination has a number of benefits. First, the use of RDF and OWL removes the technical obstacle for making models interoperable with other knowledge resources on the Semantic Web although semantic interoperability will often require an alignment process to take place for more far reaching compatibility. The modeling approach that we propose is complementary to the efforts of communities such as the Open Biomedical Ontology (OBO) community. This community's stated purpose is to create an 'accurate representation of biological reality' by developing comprehensive domain ontologies and reconciling existing ontologies according to a number of governing principles [4]. Our ambitions are more modest. We start with a minimal model to represent a hypothesis, i.e. a particular *model* of reality. We define our own classes and properties within the scope of a knowledge extraction experiment, but because of the modularity supported by OWL this does not exclude integration with other ontologies. In fact, integration with existing knowledge resources enables a complementary approach for finding facts potentially relevant to a hypothesis. Clearly, in order to scale up our methodology to represent knowledge beyond the experiments of a small group of researchers, alignment with standards would have to be considered. Upper ontologies can facilitate integration (for an example see [33]), and we can benefit from the OBO guidelines and the tools that have been developed to convert OBO ontologies to OWL [33–35]. Another interesting possibility is the integration with thesauri based on the SKOS framework [36]. Relations between SKOS concepts (terms) are defined by simple 'narrower' and 'broader' relations that turn out to be effective for human computer interfaces, and may be the best option for labeling the elements in our semantic models. Instead of providing a text string as a human readable label, we could associate an element with an entry in a SKOS thesaurus, which is a valuable knowledge resource in itself. The SKOS format is useful as an approach for 'light-weight' knowledge integration that avoids the problems of ontological over-commitment associated with more powerful logics like OWL DL [37].

A second benefit of our methodology comes from the implementation of the knowledge extraction procedure as a workflow. The procedure for populating an ontology is similar to the one previously described by Witte *et al.* [38], but our implementation allows the accumulation of knowledge by repeatedly running the same workflow or adaptations of it. This enables us to perform posterior analyses over the results from several experiments by querying the knowledge base, for instance in a new workflow that uses the AIDA semantic repository service. Moreover, the approach is not limited to text mining. If one considers text documents as a particular form of data, we can generalize the principle to any computational experiment in which the output can be related to a qualitative biological model. As such, this work extends previous work on integration of genome data via semantic annotation [39]. In this case the annotation is carried out by a workflow. Considering that there are thousands of Web Services and hundreds of workflows available for bioinformaticians [17], numerous extensions to our workflow can be explored. In addition, the combination with a semantic model allows us to collect evidence information as a type of knowledge provenance during workflow execution. In this way, we were able to address the issue of keeping a proper log of what has happened to our data during computational experimentation, analogous to the lab journal typically required in wet labs [40]. Ideally, the knowledge provenance captured in our approach would be more directly supported by existing workflow systems. However, this is not yet possible. There seems to be a knowledge gap between workflow investigators and the users from a particular application domain with regard to provenance. We propose that workflow systems take care of execution level provenance and provide an RDF interface on which users can build their own provenance model. In this context, it will be interesting to see if we will be able to replace our workflow model and link directly to the light weight provenance model that is being implemented for Taverna 2 [41]. A third benefit is that the application of Semantic Web, Web Services, and workflows stored on myExperiment.org, allow all resources relevant to an experiment to be shared on the web, making our results more reproducible. We would like to increase the 'liquidity' of knowledge so that knowledge extracted from computational experiments can eventually fit into frameworks for scientific discourse (hypotheses, research statements and questions, etc.) such as Semantic Web Applications in Neuromedicine (SWAN) [42]. If it is to be global, interoperability across modes of discourse would require large scale consensus on how to express knowledge provenance, not only about knowledge produced from computational experiments but also from manual or human assertions. Some groups are attempting to address various aspects of this problem, such as the Scientific Discourse task force [43] in the W3C Semantic Web Health Care and Life Sciences Interest Group [44], the Concept Web Alliance [45] and the Shared Names initiative [46].

## Conclusion

In this paper we demonstrate a methodology for a 'do it yourself' approach for the extraction and management of knowledge in support of generating hypotheses about biomolecular mechanisms. Our approach describes how one can create a personal model for a specific hypothesis and how a personal 'computational experiment' can be designed and executed to extract knowledge from literature and populate a knowledge base. A significant advantage of the methodology is the possibility it creates to perform analyses across the results of several of these knowledge extraction experiments. Moreover, the principle of semantic disclosure of results from a computational experiment is not limited to text mining. In principle, it can be applied to any kind of experiment of which the (interpretations of) results can be converted to semantic models, almost as a 'side effect' of the experiment at hand. Experimental data is automatically semantically annotated which makes it manageable within the context of its purpose: biological study. We consider this an intuitive and flexible way of enabling the reuse of data. With the use of Web Services from the AIDA Toolkit and others, we also demonstrated the exploitation of the expertise of computational scientists with diverse backgrounds, i.e. where knowledge sharing takes place at the level of services and qualitative models. We consider the demonstration of e-Science and Semantic Web tools for a personalized approach in the context of scientific communities to be one of the main contributions of our methodology. In summary, the methodology provides a basis for automated support for hypothesis formation in the context of experimental science. Future extensions will be driven by biological studies on specific biomolecular mechanisms such as the role of histone modifications in transcription. We also plan to evaluate general strategies for extracting novel ideas from a growing repository of structured knowledge.
